# Deep Guided Exposure Correction with Knowledge Distillation

**DOI:** 10.3390/s25247606

**Published:** 2025-12-15

**Authors:** Songrong Liu, Tao Zhang

**Affiliations:** 1 Zhejiang Communications Involvement Expressway Operation Management Co., Ltd., Hangzhou 310020, China; 2 School of Communication Engineerin, Hangzhou Dianzi University, Hangzhou 310018, China

**Keywords:** exposure correction, deep guidance, knowledge distillation

## Abstract

Images captured with unreasonable exposures will greatly reduce visual quality. Exposure problems can be categorized as follows: (i) over-exposure, i.e., bright and losing image regions caused by too-long exposure; (ii) under-exposure, i.e., dark and drowned-in noises caused by too-short exposure. Most prior works only handle over- or under-exposure problems on sRGB domain and ignore prior knowledge of channel information. In this paper, we propose **D**eep **G**uided network for exposure correction on RAW domain with **K**nowledge **D**istillation (denoted as DGKD), solving two problems together. Firstly, according to color sensitivity, we employ blue/red channel and green channel as guidance information for over- and under-exposure correction, respectively. Secondly, to handle two varying problems in a unified network, we first train the over- and under-exposure correction networks individually and then distill knowledge into one deep guided network. The experimental results show that the proposed method outperforms the state-of-the-art methods under both quantitative metrics and visual quality. Specifically, the proposed method attained a peak signal-to-noise ratio of 24.653 dB and a structural similarity index of 0.8182 on the collected RAW image exposure correction dataset.

## 1. Introduction

The exposure will affect the intensity and quality of the captured image. Modern digital cameras can control the exposure automatically with auto-exposure mode or manually by user to capture image with proper quality. However, there are several factors that lead to exposure error, including errors in measurements of through-the-lens metering, errors manually made by users, tremendous brightness changes, and difficult lighting conditions. These exposure errors occur early in image capturing and will greatly reduce the contrast and visual quality of the final image.

To solve this problem, a number of model-driven methods have been proposed for exposure correction, such as Histogram-based methods [1,2,3] and Retinex-theory-based methods [4,5]. Recently, leveraging the power of deep learning, data-driven methods [6,7,8,9,10,11] have achieved better performance, which learns nonlinear mapping function between incorrect- and proper-exposure images. Nevertheless, most methods only handle over- or under-exposure problems. It limits real-world applications of correcting various exposures.

Accordingly, more and more studies [12,13,14] pay attention to solving over- and under-exposure correction problem in an end-to-end network. However, these methods either ignore the requirement of different prior knowledge learning or design a simple module to implicitly learn various prior knowledge for over- and under-exposure correction. Due to significantly different correction procedures of over- and under-exposure, correcting only over- or under-exposure is varying. Thus, it is a challenge to simultaneously correct both unreasonable exposures with a single network. The key to solving this problem is how to effectively learn different prior knowledge in a single network.

Additionally, most of the previous methods perform exposure correction on 8-bit standard (sRGB) images, rendered from RAW data by highly nonlinear operations, i.e., camera image signal processing (ISP). Due to compression and quantization routines of ISP, sRGB has information lost, leading to a potential bottleneck for exposure correction. In fact, previous works [7] show that RAW data contains more abundant information with higher bit value and preserves the linear relationship between exposure and scene radiance, which benefits from image restoration.

**Figure 1:**
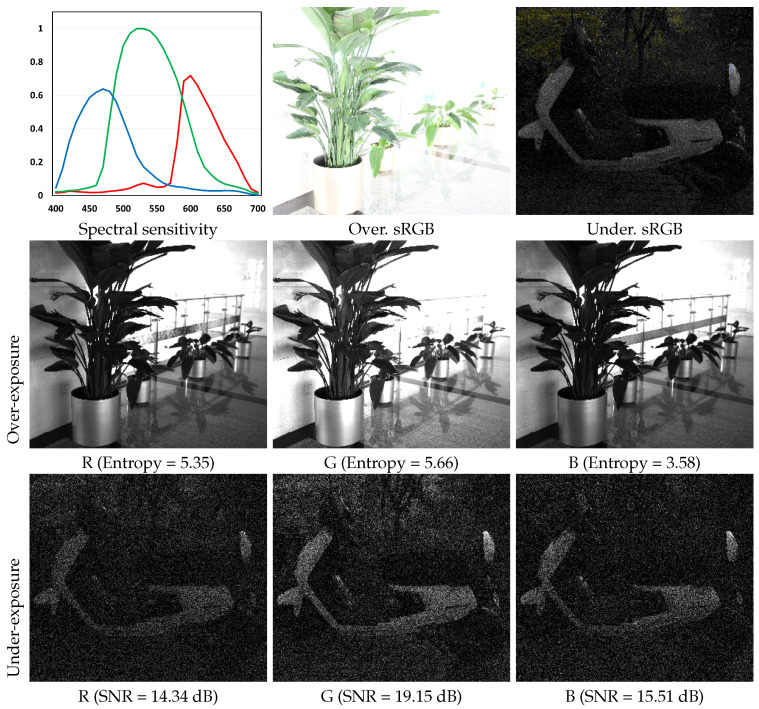
Comparison of different color channels for over- and under-exposure images. Over: sRGB means over-exposure sRGB image, which is bright and losing image regions. Under: sRGB means under-exposure sRGB image, which is dark and has drowned-in noises. Camera spectral sensitivity of green is higher than that of red and blue, which leads to higher intensity. For over-exposure, the information entropy of the green channel is lower than that of red and blue channels. For under-exposure, the SNR of the green channel is higher than that of others. Note that we only show one green channel of the RAW data and the other is similar to the one shown.

In this paper, we propose a deep guided network for exposure correction on RAW domain with knowledge distillation, which learns different prior knowledge for over- and under-exposure correction. To this end, we present color channel guidance and knowledge distillation. For the former, we recognize that most digital camera sensors are designed to have higher sensitivity of green channel than red and blue channels, as human eyes are more sensitive to green than others. As shown in Figure 1, we can observe that green channels are brighter than others and more likely to lose information from over-exposure while having higher signal-to-noise for under-exposure. It means that red and blue channels for over-exposure correction and green channels for under-exposure correction are easier to be restored. Motivated by this, we design a color channel guidance branch and employ red/blue channels and green channels for over- and under-exposure correction, respectively. Moreover, we design a guided channel selection module to automatically select desired channels for the corresponding corrections. For the latter, the exposure correction model should be able to tackle over- and under-exposure problems without additional computational cost while achieving proper correction performance. In general, it is difficult to train a single network for various tasks. Fortunately, only over- and under-exposure corrections are two simpler tasks and can learn prior knowledge more effectively. Motivated by this, we first individually train the over- and under-exposure correction networks to learn the specific prior knowledge, and employ knowledge distillation to distill different prior knowledge to a single network and ensure that the guided channel selection module selected the proper channels. Additionally, to support the exposure correction in the real world, we collect a real RAW dataset with paired incorrect and proper-exposure images. The experimental results show that the proposed method outperforms the state-of-the-art methods under both objective quantitative metrics and subjective visual quality.

Main contributions can be summarized as follows:

- We present a deep guided exposure correction method, which can learn different prior knowledge for over- and under-exposure correction at the same time.
- We design a deep guided network to automatically exploit color guidance information by considering the sensitive property of different channels.
- We introduce a knowledge distillation strategy to effectively learn a unified network by distilling varying well-learned prior knowledge.

For over-exposure, some information is clipped and lost, and the information entropy can measure the information content in a clipped image.

For under-exposure, the image is drawn in noise, and the SNR can measure the quality of a noisy image.

## 2. Related Work

**Model-driven Image Exposure Correction Methods.** Exposure correction aims to obtain proper-exposure images from over- or under-exposure images [15,16]. Histogram-based methods [1,2,3] mapped the histogram to improve image contrast. Among these methods, Reza [17] corrected exposure by adaptively adjusting the histogram of varying image regions. Tian et al. [3] enhanced low-contrast images by combining global and local histogram mapping. Retinex-theory [18] is another mostly used technique for exposure correction, which decomposed an image into reflectance and illumination components, and enhanced the latter component. Fu et al. [4] combined multiple derivatives of an estimated illumination to generate a high-contrast image. Guo et al. [5] leveraged maximum intensity of RGB channels as the initial coarse illumination map, which was then refined with a structure prior. Zhang et al. [19] optimized constrained illumination estimation with perceptually bidirectional similarity to adjust the brightness. Li et al. [20] designed objective function with an additional noise term and employed an optimization technique to resolve it. However, most algorithms rely on hand-crafted priors to estimate and adjust brightness, which introduces undesired artifacts.

**Data-driven Image Exposure Correction Methods.** Recently, data-driven algorithms [6,7,21,22,23,24,25,26,27] have been proposed to automatically learn the desired prior for image restoration. Most data-driven exposure correction methods focus on under-exposure correction. Retinex theory is employed by a number of these methods. Wei et al. [8] adjusted the illumination in a data-driven manner, and Zhang et al. [28] further recovered the reflectance component by a sub-network. As another form of component decomposition, Yang et al. [9] employed a recursive learning method to enhance the representations of different bands for both paired and unpaired datasets. Ren et al. [29] proposed a two-stream network to simultaneously learn global information and salient structures in clear images. Moreover, some methods are based on self-supervised techniques [10]. Guo et al. [10] formulated exposure correction as a image-specific curve estimation task with a self-supervised network. Recently, some methods are proposed to tackle non-uniform exposure correction. For instance, Lv et al. [11] first fused the over- and under-exposure corrected results, and then they refined the details with a lightweight network for non-uniform illumination image enhancement.

Apart from these methods, more and more works have been proposed to correct over- and under-exposure images simultaneously. Ma et al. [12] first handled both over- and under-exposure by a shared-weight network, before fusing them to obtain an image with accurate exposure correction. Afifi et al. [13] proposed a coarse-to-fine network with Laplace pyramid features to correct exposures, which first restored the brightness and then refined the details. More recently, Huang et al. [14] employed exposure consistency learning to consistently learn the representations of both over- and under-exposure in the feature space. Zhang et al. [30] utilized a illumination-guided dual-branch fusion network for partition-based image exposure correction. These methods either ignore the requirement of different prior knowledge learning or design a simple module to implicitly learn various prior knowledge for over- and under-exposure correction. In this work, we propose a deep guided network to learn different prior knowledge for over- and under-exposure correction.

**Guided Image Recovery.** Guided image recovery utilizes additional assistant information for image restoration. The well-known guided filter [31] employs image guidance to generate filter weights and has shown its effectiveness in various tasks of image restoration. Recently, deep learning methods [32,33,34,35,36] utilize varying information as guidance for various image recovery tasks. A number of methods [32,37] utilized RGB image to assist the super-resolution of the depth image. Zhou et al. [35] proposed a guided super-resolution model with cross-scale stereo image as a guide. Fu et al. [36] utilized RGB image as a guiding image for hyperspectral image super-resolution. Zhang et al. [38] employed channel information to guide image denoising and demosaicing. In addition, Gu et al. [39] proposed a self-guidance network for image denoising, where the features in low resolution were employed to guide the high-resolution features. Liu et al. [40] developed a green channel guidance network for joint denoising and demosaicing by considering the high sampling rate of the green channel.

These methods employ the high-SNR property of the green channel in under-exposure to guide image recovery, but they ignore the high-entropy characteristic of red and blue channels in over-exposure. Thus, we present a deep guided network for exposure correction, which can automatically select the desired guidance channel information and assist in correcting image capture under both over- and under-exposure conditions.

**Knowledge Distillation.** Knowledge distillation employs a teacher–student architecture to transfer the knowledge from one model to anther. The original technique [41] employed the soft probabilities of a larger teacher model to supervise a smaller student network. This idea was extended by [42], which employed the intermediate feature maps in the teacher to assist the training process of the student network. The knowledge distillation has achieved satisfactory results in various high-level vision tasks, such as image classification [43], object detection [44], semantic segmentation [45], and so on.

Apart from high-level vision tasks, knowledge distillation have been utilized for image restoration [46,47]. Zhou et al. [46] employed dynamic contrastive knowledge distillation for image restoration. Jiang et al. [47] utilized multi-teacher knowledge distillation for image super-resolution. These methods conduct several sub-tasks to explore various prior knowledge, and distill them into the final image restoration network. Over-exposure correction and under-exposure correction are naturally two sub-tasks of exposure correction. Therefore, we employ knowledge distillation for exposure correction, which can effectively distill the specific knowledge of over- and under-exposure correction into a unified exposure correction model.

## 3. Method

In this section, we first introduce the motivation of the proposed method. Then, we describe the architecture of deep guided network, which can learn different prior knowledge for over- and under-exposure correction. Furthermore, we introduce the color channel guidance and guided channel selection modules, which can consider the sensitive property of different color channels and automatically select the desired channel for the corresponding correction. Finally, the knowledge distillation strategy is described, which can distill the well-learned knowledge of over- and under-exposure to a unified exposure correction network.

### 3.1. Motivation

The goal of exposure correction is to transform the over- and under-exposure images into proper-exposure images. Previous methods [12,13,14] usually employ one network to handle over- and under-exposure images simultaneously. However, the correction procedures of over- and under-exposure are significantly different, and the required prior knowledge of them are obviously different. In addition, previous methods perform exposure correction on sRGB image, which has been nonlinearly processed by ISP. On the contrary, the RAW image is linearly related to real-world signals and has more information [7]. Thus, it is reasonable to learn varying prior knowledge for over- and under-exposure on the RAW image.

To model the working mechanism of human eyes, the consumptive digital camera is always designed to have higher relative spectral sensitivity to the color green, due to its higher perceptual intensity [48]. Combined with the observation of real-world over- and under-exposure RAW images of Canon EOS 5D Mark IV, as shown in Figure 1, we find that the green channel has higher intensity, leading to easier loss of information for over-exposure while having higher signal-to-noise for under-exposure. It reveals that the red and blue channels for over-exposure correction and green channels for under-exposure correction are easier to be restored. Furthermore, the exposure correction model should be able to tackle over- and under-exposure problems without additional computational cost while also achieving proper correction performance. It is a challenge to train a network for various tasks, i.e., over- and under-exposure correction. A previous method [49] shows that knowledge distillation can distill different prior knowledge into a unified network.

Accordingly, we design a deep guided network and a knowledge distillation strategy, and directly employ RAW image as an input to estimate proper-exposure images. Specifically, the deep guided network can automatically select the desired channels and employ them as the guidance (or condition) information for both over- and under-exposure correction. The knowledge distillation can effectively distill the different well-learned prior knowledge of over- and under-exposure correction into a unified network.

### 3.2. Deep Guided Network

**Figure 2:**
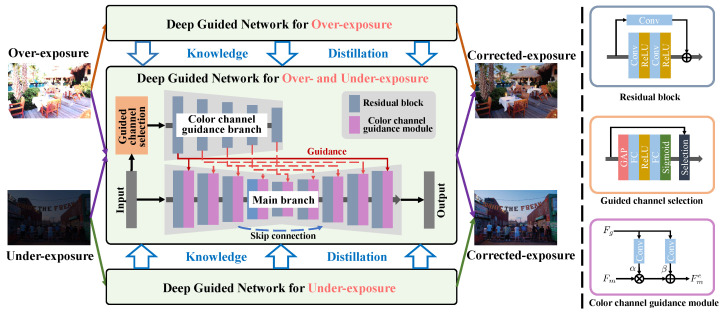
The overview of the proposed method, including deep guided network and knowledge distillation. The deep guided network architecture consists of the main branch, the color channel guidance branch, and the guided channel selection module. The guided channel selection module automatically select the desired color channels for over- or under- exposure correction. The color channel guidance branch employs them to generate guidance features, which are utilized to modulate the corresponding features in the main branch. The network architecture of the guidance branch is the same as the encoder of the main branch, except with fewer feature maps. The knowledge distillation involves distilling the well-learned prior knowledge of over- and under-exposure correction into a unified deep guided network.

The architecture of guided network is illustrated in Figure 2. The overall structure consists of three parts, including the main branch, the color channel guidance branch, and the guided channel selection module. To fully utilize the useful information within the input RAW image, we decompose it into RGGB four channels. The red and blue channels have richer information for over-exposure correction, while the two green channels are beneficial for under-exposure correction. As different guidance channels assist in varying exposure corrections, we first employ the guided channel selection module to automatically select the desired color channels for over- or under-exposure correction. Then, we feed the selected color channels into the color channel guidance branch to generate guidance features. Finally, we employ the guidance features to modulate the corresponding features in the main branch. This way, the guidance information is automatically selected and effectively employed for over- and under-exposure correction, respectively.

The main branch is based on typical Unet [50] architecture. It consists of 4 encoder stages and 4 corresponding decoder stages. After each encoder stage, we employ 4×4 kernel size convolution with 2 strides to downsample the feature. At the begin of each decoder stage, we utilize a transposed convolution to upsample the feature maps. Skip connections are employed to connect large-scale low-level and high-level feature maps in each encoder stage and the corresponding decoder stage. To ease the training, residual block is utilized as the basic block to constitute encoder and decoder. The residual block consists of a 1×1 convolution and two 3×3 convolutions followed by ReLU activation function.

**Guided Channel Selection.** To automatically select the desired channels for over- and under-exposure correction, we design a guided channel selection module, as shown in the center-right of Figure 2. We first feed the input the four-channel RAW image into a global average pooling (GAP) layer to obtain the mean vector. Subsequently, in order to further obtain the weight information of varying channels, two fully connected (FC) layers are employed to learn the nonlinear relationship between each channel. Then, we utilize Sigmoid gating mechanism to limit the final attention weight of each channel. The corresponding channels of two highest values is the selected channels.

As analyzed above, the red and blue channels are easier to be restored for over-exposure and green channels are restored for under-exposure. Inspired by it, when training the network for over- and under-exposure correction, we constrain the guided channel selection module to select specific channels for corresponding correction task. It can be expressed as

(1) $$L_{s}=\left|\right|I_{s}-I_{d}{\left|\right|}_{1},$$

where $I_{s}$ is the selected channels and $I_{d}$ is the desired channels, i.e., blue and red channels for over-exposure correction and green channels for under-exposure correction.

**Color Channel Guidance.** To make the main branch features suitable for different exposure corrections, we design a color channel guidance module, as shown in the right bottom of Figure 2. Inspired by [31,51], the color channel guidance module modulates the features by the guidance information. Firstly, we feed the selected color channels in to the color channel guidance branch to generate guidance features. Then, the guidance feature with corresponding spatial resolution is employed to modulate the main branch feature. Specifically, we utilize two convolution layers to generate pixel-wise scaling and bias from the guidance feature $F_{g}$, which are employed to enhance the main branch feature $F_{m}$. It can be represented as

(2) $$F_{m}^{e}=\alpha \left(F_{g}\right)\cdot F_{m}+\beta \left(F_{g}\right),$$

where $F_{m}^{e}$ is the enhanced feature. $\alpha (F_{g})$ and $\beta (F_{g})$ are two learned modulation parameters.

### 3.3. Knowledge Distillation

To effectively learn various prior knowledge in a unified network, we employ knowledge distillation for over- and under-exposure correction, as shown in the left top and bottom of Figure 2. Before knowledge distillation, we first train two specific networks for over- and under-exposure correction. The learning function can be expressed as

(3) $$L=L_{s}+L_{r},$$

where $L_{s}$ and $L_{r}$ are guided color selection loss and reconstruction loss, respectively. The reconstruction loss can be represented as

(4) $$L_{r}=||O-{T||}_{1},$$

where *O* and *T* are the output of exposure correction network and the corresponding ground-truth, respectively.

When the over- and under-exposure correction networks are well trained, we employ knowledge distillation to distill the knowledge for over- and under-exposure correction into an unified network. Specifically, we utilize the feature distillation technique. For feature distillation, the two well-trained over- and under-exposure correction networks are the teacher network, and the unified over- and under-exposure correction networks is the student network. The features in student network should be close to the corresponding features in the teacher networks for over- and under-exposure correction, respectively. The procedure of knowledge distillation can be expressed as

(5) $$L=L_{s}+L_{r}+L_{kd},$$

where $L_{kd}$ is the loss between features of teacher and student network. It can be represented as

(6) $$L_{kd}=\sum_{i=1}^{N}\lambda_{i}\left|\right|F_{s}^{i}-F_{t}^{i}{\left|\right|}_{1},$$

where *N* is the number of used features in deep guided network, $\lambda_{i}$ is the hyper-parameter, $F_{s}^{i}$ and $F_{t}^{i}$ are the features of student and teacher networks, respectively. $F_{t}^{i}$ can be the feature in over- or under-exposure correction networks, and is utilized to supervise $F_{s}^{i}$ in the unified network.

## 4. Experiments

In this section, we first describe the datasets and settings employed in our experiments. Then, we provide the comparison results of our method and several state-of-the-art methods. Finally, we discuss the effectiveness of different modules, knowledge distillation, image domain, and cross sensors.

### 4.1. Dataset

Previous data-driven exposure correction methods [12,13,14] are performed on synthetic sRGB images under a simple exposure setting. For example, the dataset in [13] only contains sRGB images rendered from RAW images. The sRGB image is nonlinearly processed and has lower bit range, which limits the performance of the exposure correction network in correcting various exposures in the real world [52,53].

To support the exposure correction on RAW images, we conduct a real RAW exposure correction dataset with paired incorrect- and proper-exposure RAW images. To capture the dataset, we employ a Canon EOS 5D Mark IV camera. The camera is mounted on sturdy tripods and is controlled by a remote software. We first adjust camera settings such as focus, exposure time, and aperture to maximize the quality of the ground-truth images for each scene. Then, we adjust the exposure time with a factor from 1/100 to 10 to capture under- or over-exposure images. Due to multiple acquisitions of one scene, all scenes in the dataset are static. Our dataset contains 4830 paired RAW images with 6744×4502 spatial resolution.

### 4.2. Settings

**Implementation Details.** To train the proposed method, we randomly crop overlapped patches with 256×256 spatial resolution from images in our paired real exposure correction dataset. We employ PyTorch [54] to implement the proposed method. In each training epoch, we employ Adam optimizer [55] ($\beta_{1}=0.9$ and $\beta_{2}=0.999$) to optimize the network. We set the initial learning rate and mini-batch size as $1\times 10^{-4}$ and 1, respectively.

**Competing Methods.** We compare our method (deep guided network with knowledge distillation, DGKD) with six state-of-the-art methods, including Zero-DCE [10], RUAS [56], MSEC [13], ECCM [57], ECLNet [14], and IDFN [30]. We utilize the official codes of compared methods and retrain them with the same setting of our method on our captured real RAW exposure correction dataset.

**Evaluation Metrics.** We employ two metrics to evaluate the performance of all methods, including the peak signal-to-noise ratio (PSNR) and structural similarity (SSIM). The larger PSNR and SSIM indicate better performance.

### 4.3. Evaluation of Exposure Correction

**Quantitative Results.** Table 1 provides the averaged recovery results of different cases on our real RAW exposure correction dataset to quantitatively compare our method with Zero-DCE, RUAS, MSEC, ECCM, ECLNet, and IDFN. The best results are highlighted in bold for each metric. Due to varying difficulties of different exposure corrections, over-exposure correction achieves higher PSNR and SSIM than under-exposure correction. It can be seen that MSEC, ECCM, ECLNet, IDFN, and our method achieve better performance than Zero-DCE and RUAS, as they are not specifically designed for simultaneous over- and under-exposure correction. Among the specific exposure correction methods, our method outperforms MSEC, ECCM, ECLNet, and IDFN. It demonstrates the effectiveness of the proposed method, which can learn different prior knowledge in a unified network. Moreover, the inference time of our method is comparable with Zero-DCE, MSEC, and IDFN, and it is faster than RUAS, ECCM, and ECLNet. Our method can achieve competing inference time with better performance.

**Figure 3:**
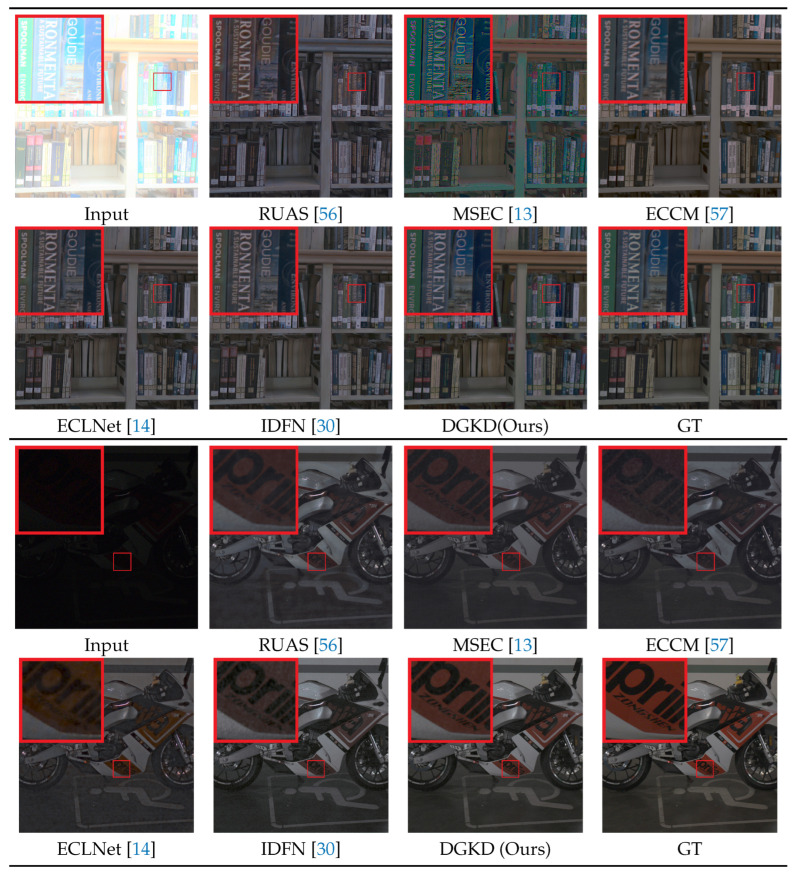
Visual quality comparison on two typical scenes for over- and under-exposure correction in our real dataset. The top is the over-exposure correction results and the bottom is under-exposure. For each scene, the results recovered by different methods and the ground-truth image are shown from left to right and from top to bottom.

**Perceptual Quality.** To visualize the experimental results, two representative corrected results for over- and under-exposure correction are shown in Figure 3. The input, recovered results of RUAS, MSEC, ECCM, ECLNet, IDFN and our method, along with ground-truth, are shown from left to right and from top to bottom. We can see that our method has better visual results than the compared methods. For over-exposure, RUAS and ECCM cannot well-recover the color. The result of MSEC is still a bit over-exposed. There are undesired artifacts in the results of ECLNet and IDFN. For under-exposure, there still have noise in the results of MSEC, ECCM, and IFDN. RUSA and ECLNet do not denoise well, and there are some artifacts. It demonstrates the effectiveness of the proposed method.

### 4.4. Discussion

Here, we discuss the effect of different network modules, knowledge distillation, image domain, and cross sensors.

**Figure 4:**
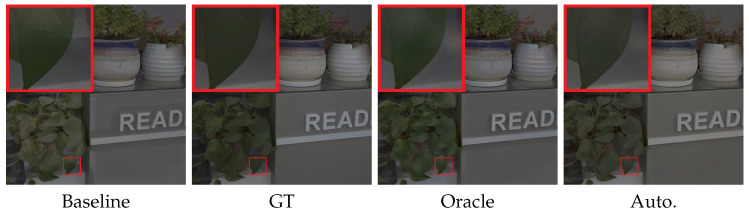
Visual quality comparison for different modules.

**The Effect of Different Network Modules.** To investigate the effectiveness of the color channel guidance and guided color selection, we conduct an ablation study on real exposure correction dataset. The results are provided in Table 2. We can see that the proposed color channel guidance (Oracle and Auto.) can effectively improve the performance of both over- and under-exposure correction. It verifies the effectiveness of color channel guidance, which can guide the learning of different prior knowledge for over- and under-exposure correction. In addition, automatic color channel guidance (i.e., color channel automatically selected by guided channel selection module) can achieve similar performance with oracle color channel guidance (i.e., red and blue channels for over-exposure and green channels for under-exposure). It demonstrates that guided channel selection module can automatically select the desired guidance channel for both over- and under-exposure correction. Moreover, we show the visual results of the proposed method with different network modules in Figure 4. We can see that there are undesired artifacts in results of the baseline network, and our method with oracle and automatic color guidance achieves comparable results. It further shows the effectiveness of color channel guidance and automatic guided channel selection.

**The Effect of Knowledge Distillation.** To verify the effectiveness of knowledge distillation, we compare our method with a variation of our method without employing knowledge distillation and networks separately trained for over- and under-exposure correction. The results are provided in Table 3. It can be seen that knowledge distillation can improve the performance of unified network for both exposure corrections, which effectively distills the specific knowledge of over- and under-exposure correction into a unified model. Moreover, with knowledge distillation, the proposed deep guided network can achieve similar performance with the severally trained models for each exposure correction. It verifies the effectiveness of knowledge distillation for exposure correction.

**The Effect of Image Domain.** To evaluate the effect of image domains, we compare our method on sRGB-to-sRGB (S2S), RAW-to-sRGB (R2S), and RAW-to-RAW (R2R) domains. The results are provided in Table 4. We evaluate all models in sRGB domain for fair comparison. Note that the sRGB of RAW-to-RAW model is generated by feeding the output of the deep guided network into the same ISP of ground-truth. We can see that the performance of S2S is significantly worse than R2S and R2R. The reason is that the sRGB input is nonlinearly processed and is in 8-bit data range, where the information is heavily compressed and lost. It means the RAW input domain is very important for exposure correction. Further, compared to R2S, R2R achieves visible improvement for avoiding the inconsistent ISP processes, e.g., white balance, for different scenes. The R2R handles the most essence problem and ignores the important aspects of exposure correction. It shows the benefits of exposure correction in R2R image domain.

**Figure 5:**
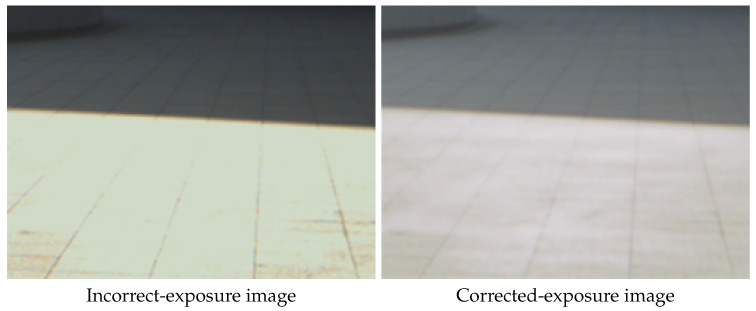
Generalization to Sony IMX-serial sensor.

**The Effect of Cross Sensors.** To evaluate the effect of generalization cross different sensors, we employ a mobile phone with Sony IMX-serial sensor to capture RAW image and utilize our method to correct the exposure. The visual results are shown in Figure 5. We can see that the proposed method can correct image captured by other sensors, which demonstrate the generalization ability of the proposed method.

## 5. Conclusions

In this paper, we propose a novel guided network with knowledge distillation for exposure correction on RAW images. The guided network adaptively selects the desired color channels and employs them as guidance information to learn different prior knowledge of over- and under-exposure correction. The knowledge distillation distills the different prior knowledge into an unified network. In addition, to facilitate the studies on RAW image exposure correction, we collect a paired real RAW dataset with both over- and under-exposure images. Experimental results show that the proposed method outperforms current state-of-the-art methods under both comprehensive quantitative metrics and perceptive quality. In the future, we will further consider the effective guidance information, e.g., segmentation of over-exposed region and noise map of under-exposure images, to assist exposure correction network in learning different prior knowledge in a unified network.

## Figures and Tables

**Table 1:** Quantitative results of different methods on our real exposure correction dataset. The best results are highlighted in **bold**.

Cases	Metrics	Zero-DCE	RUAS	MSEC	ECCM	ECLNet	IDFN	DGKD
		[10]	[56]	[13]	[57]	[57]	[30]	(Ours)
Over	PSNR	20.215	25.349	20.812	27.118	27.641	27.634	**27.828**
	SSIM	0.8382	0.9243	0.8991	0.9371	0.9432	0.9402	**0.9505**
Under	PSNR	15.830	19.231	20.038	20.334	20.674	21.057	**21.479**
	SSIM	0.4103	0.6407	0.6507	0.6676	0.6788	0.6791	**0.6859**
Average	PSNR	18.022	22.290	20.425	23.726	24.158	24.345	**24.653**
	SSIM	0.6243	0.7825	0.7749	0.8024	0.8110	0.8097	**0.8182**
Inference Time (ms)		2.92	14.64	5.77	14.83	18.54	9.25	9.18

**Table 2:** The effect of different network modules. Oracle denotes directly employing red and blue channels for over-exposure and green channels for under-exposure. Auto. is utilizing the channels automatically selected by color channel selection module.

Cases	Metrics	Baseline	+Color Guidance	
			Oracle	Auto.
Over	PSNR	27.009	27.867	27.828
	SSIM	0.9367	0.9508	0.9505
Under	PSNR	20.293	21.485	21.479
	SSIM	0.6783	0.6862	0.6859
Average	PSNR	23.651	24.676	24.653
	SSIM	0.8075	0.8185	0.8182

**Table 3:** The effect of different learning strategies. KD denotes knowledge distillation.

Cases	Metrics	Separated	Unified	
			w/o KD	w/KD
Over	PSNR	27.934	27.671	27.828
	SSIM	0.9535	0.9431	0.9505
Under	PSNR	21.618	21.125	21.479
	SSIM	0.6897	0.6807	0.6859
Average	PSNR	24.776	24.398	24.653
	SSIM	0.8216	0.8119	0.8182

**Table 4:** The effect of different image domain. S2S, R2S, and R2R denote sRGB-to-sRGB, RAW-to-sRGB, and RAW-to-RAW.

Cases	Metrics	S2S	R2S	R2R
Over	PSNR	23.589	27.542	27.828
	SSIM	0.8834	0.9418	0.9505
Under	PSNR	17.867	20.594	21.479
	SSIM	0.6213	0.6808	0.6859
Average	PSNR	20.728	24.068	24.653
	SSIM	0.7524	0.8113	0.8182

## Data Availability

Due to the company’s requirements for data privacy, we are unable to disclose the dataset publicly.
